# Recombination of a dual-CAR-modified T lymphocyte to accurately eliminate pancreatic malignancy

**DOI:** 10.1186/s13045-018-0646-9

**Published:** 2018-08-13

**Authors:** Erhao Zhang, Peiwei Yang, Jieyi Gu, Heming Wu, Xiaowei Chi, Chen Liu, Ying Wang, Jianpeng Xue, Weiyan Qi, Qingbo Sun, Shengnan Zhang, Jialiang Hu, Hanmei Xu

**Affiliations:** 1 The Engineering Research Center of Peptide Drug Discovery and Development, China Pharmaceutical University, Nanjing, 210009 People’s Republic of China; 2 Basic Medical Research Center, School of Medicine, Nantong University, Nantong, 226001 People’s Republic of China; 3 Jiangsu Key Laboratory of Oral Diseases, Department of Oral and Maxillofacial Surgery, Affiliated Hospital of Stomatology, Nanjing Medical University, Nanjing, 211166 People’s Republic of China; 4 State Key Laboratory of Natural Medicines, Ministry of Education, China Pharmaceutical University, Nanjing, 210009 People’s Republic of China; 5 Nanjing Anji Biotechnology Co., Ltd, Nanjing, 210046 People’s Republic of China

**Keywords:** Cancer immunotherapy, Dual-receptor CAR, Pancreatic cancer, Carcino-embryonic antigen, Mesothelin

## Abstract

**Background:**

The therapeutic application of T cells endowing with chimeric antigen receptors (CARs) is faced with “on-target, off-tumor” toxicity against solid tumors, particularly in the treatment of the pancreatic cancer. To our best knowledge, the pancreatic cancer cell line AsPC-1 often highly expressed some distinct tumor-associated antigens, such as carcino-embryonic antigen (CEA) and mesothelin (MSLN). Therefore, in this research, we have characterized dual-receptor CAR-modified T cells (dCAR-T) that exert effective and safe cytotoxicity against AsPC-1 cells.

**Methods:**

Based on the dual signaling pathway of wild T cells, we designed a novel dCAR diagram specific for CEA and MSLN, which achieved comparable activity relative to that of conventional CAR-T cells (CEA-CAR T or MSLN-CAR T). In this dCAR, a tandem construct containing two physically separate structures, CEA-CD3ζ and MSLN-4/1BB signaling domains were effectively controlled with tumor antigens CEA and MSLN, respectively. Finally, the activity of dCAR-T cells has been verified via in vitro and in vivo experiments.

**Results:**

In the presence of cognate tumor cells (AsPC-1) expressing both CEA and MSLN, dCAR-T cells exerted high anti-tumor activity relative to that of other single-receptor CAR-T cells bearing only one signaling pathway (e.g., Cζ-CAR and MBB-CAR). In a xenograft model, dCAR-T cells significantly inhibited the growth of AsPC-1 cells yet no effect on the growth of non-cognate tumor cells. Furthermore, the released cytokines and T cell persistence in mice were comparable with that of conventional CAR-T cells, obtaining specific and controllable cytotoxicity.

**Conclusions:**

A novel type of CAR-T cells, termed dCAR-T, was designed with specific activities, that is, significant cytotoxicity for two antigen-positive tumor cells yet no cytotoxicity for single antigen-positive tumor cells. Dual-targeted CAR-T cells can be precisely localized at the tumor site and can exert high cytotoxicity against tumor cells, alleviating “on-target, off-tumor” toxicity and enabling accurate application of CAR-T cell therapy.

**Electronic supplementary material:**

The online version of this article (10.1186/s13045-018-0646-9) contains supplementary material, which is available to authorized users.

## Background

Chimeric antigen receptor (CAR) T cell therapy is a cancer treatment that uses a patient’s T cells modified to express specific proteins allowing T cells to better recognize cancer cells as well as become highly activated to exert cytotoxicity against tumors [1–4]. Once in the body, this type of cancer immunotherapy is capable of immediately providing ongoing tumor control and possible protection against recurrence in some clinical trials, a promising approach to adoptive T cell therapy for cancer treatment [5–9]. Although this type of immunotherapy holds a tremendous response for tumor elimination, especially in some hematological malignancies [10–12], it has recently been faced with a serious limitation caused by treatment-induced adverse effects in some clinical trials, such as “on-target, off-tumor” toxicity [13, 14]. In general, this toxicity derived from CAR-T cell therapy can lead to killing of non-tumor cells as a result of tumor-associated antigens expressed on normal tissues [15–17]. For example, infusion of CD19-specific CAR-T cells in one patient with B cell malignancies often caused long-term B cell aplasia symptoms, resulting from eradication of normal B cells [18, 19]. For another case, after infusion of T cells endowed with a CAR specific for human epidermal growth factor receptor 2 (HER-2), a patient with the breast cancer underwent lethal inflammatory cytokine release due to HER-2 expression on lung epithelial cells [20]. Therefore, some new approaches to treat solid tumors with sustained tumor elimination and reduced side effects are needed.

Pancreatic cancer with a metastatic property is the most lethal human cancer with poor prognosis, which the average 5-year survival rate is only 5%, with standard treatment based on surgery, radiotherapy, and chemotherapy promoting an overall survival of about 6 months, highlighting the need for more effective treatments [21–23]. Cancer immunotherapy involving T cells with redirected specificity via expression of CAR may complement such treatments, resulting in complete elimination. To further improve safety and efficacy of CAR-T cell therapy for pancreatic cancer treatment, here, we designed a novel CAR structure to render engineered T cells specific for pancreatic cancer cells in an antigen-dependent manner. Based on the dual signaling pathway of natural T cells, the dual-targeting CAR facilitates T cell immune response against dual antigen-expressing tumors compared to single-positive tumors, resulting from the two signaling domains of CAR structure controlled by the distinct tumor antigens. To our best knowledge, some previous papers reported that carcino-embryonic antigen (CEA) and mesothelin (MSLN) are tumor-associated antigens (TAAs), which are simultaneously overexpressed in the majority of pancreatic cancers, ovarian cancers, and other cancers, resulting in the pancreatic cancer AsPC-1 expressing MSLN and CEA as a cognate tumor cell in this work [24–27]. Theoretically, in this study, engineering T cells with a dual-receptor chimeric antigen receptor (dCAR) requires CAR-mediated recognition of one antigen, here being CEA, for activation, and then, their co-stimulation must be mediated by the other CAR engaging a second antigen, which is MSLN.

Herein, we demonstrate, for the first time to our knowledge, that dCAR-modified T cell exerts effective and safe cytotoxicity for the treatment of pancreatic cancer cell expressing the tumor antigens CEA and MSLN in vitro and in vivo. In this study, we proposed a novel CAR-T cell therapy that not only discriminates tumor tissues and normal tissues, but also promotes effector cell localization, thereby preventing the tumor escape from immune surveillance. Compared with the single-positive antigen-expressing tumor, T cells endowing with a dCAR could eradicate some tumors that express two antigens. Finally, the “two tumor antigen-input” strategy may accurately control the activity of dCAR-T cells, which ultimately helps broaden the applicability of this cancer immunotherapy and decrease some serious side effects of CAR-modified T cell therapies in the field of solid tumor treatment.

## Methods

### Cell lines and culture conditions

Fresh blood was obtained from healthy volunteers after informed consent on protocols was approved by the China Pharmaceutical University Institutional Review Board. Peripheral blood mononuclear cells (PBMCs) were purified from the blood by gradient centrifugation using Lymphoprep™ (Axis-Shield), and then, human T cell subtypes (i.e., CD4^+^ T cells and CD8^+^ T cells) were enriched by positive selection using the magnetic bead separation (Miltenyi Biotec). Isolated T cells were cultured in X-VIVO15 medium (Lonza) supplemented with 5% human AB serum (vol/vol, Valley Biomedical Inc.), and 10 mM *N*-acetyl l-Cysteine (Sigma-Aldrich). Lastly, the T cell medium was supplemented with 30 IU/mL or 100 IU/mL human IL-2 (PeproTech) for CD4^+^ T cell or CD8^+^ T cell growth, respectively.

The pancreatic cancer cell line, AsPC-1 cells and PANC-1 cells, the colorectal cancer cell line, HT29 cells, and the glioma cell line, U87 cells, were obtained from the American Type Culture Collection (ATCC). AsPC-1 cells were cultured in RPMI 1640-media (Hyclone), while PANC-1 cells, HT29 cells, and U87 cells were grown in Dulbecco’s modified Eagle’s medium (DMEM) media (Hyclone). All of the tumor cell culture media were supplemented with 10% fetal bovine serum (FBS), 2 mmol/l-glutamine (Gibco), 100 U/mL penicillin, and 100 μg/mL streptomycin (Sangong Biotech).

Cell line 293 T was also acquired from ATCC and maintained in DMEM medium supplemented with 10% FBS, 10 mM HEPES (Gibco), 2 mmol/l-glutamine, 100 U/mL penicillin, and 100 μg/mL streptomycin. All cell lines were incubated at 37 °C in a humidified atmosphere with 5% carbon dioxide.

### Construction of plasmids

The lentiviral vector encoding various CARs or red fluorescence protein (RFP) was constructed based on the pLV-puro vector (Hanbio Biotechnology Co., Ltd.). Briefly, for modifying T cells, the dCAR vector consisted of the following components in frame from 5′ end to 3′ end: a *Xho*Isite, Kozak and signaling peptide sequences, anti-MSLN scFv, hinge and transmembrane of CD8α molecule, cytoplasmic domain of 4/1BB, internal ribosome entry site (IRES) sequence and signaling peptide sequence, anti-CEA scFv, hinge and transmembrane of CD8α molecule, cytoplasmic domain of CD3ζ, P2A and green fluorescence protein (GFP) sequences, and a *Xba*Isite. The Cζ-CAR plasmid included some gene elements as follows (5′ end to 3′ end): a *Xho*Isite, Kozak and signaling peptide sequences, anti-CEA scFv, hinge and transmembrane of CD8α molecule, cytoplasmic domain of CD3ζ, P2A and GFP sequences, and a *Xba*Isite. The MBB-CAR plasmid included some gene elements as follows (5′ end to 3′ end): a *Xho*Isite, Kozak and signaling peptide sequences, anti-MSLN scFv, hinge and transmembrane of CD8α molecule, cytoplasmic domain of 4/1BB, P2A and GFP sequences, and a *Xba*Isite. The second-generation CAR plasmids, CEA-CAR or MSLN-CAR, had some gene elements as follows (5′ end to 3′ end): a *Xho*Isite, Kozak and signaling peptide sequences, anti-CEA scFv or anti-MSLN scFv, hinge and transmembrane of CD8α molecule, cytoplasmic domains of 4/1BB and CD3ζ, P2A and GFP sequences, and a *Xba*Isite. For modifying target cells, the RFP vector included the following components (5′ end to 3′ end): a *Xho*Isite, Kozak and signaling peptide sequences, RFP sequence, and a *Xba*Isite.

The anti-MSLN scFv portion or anti-CEA scFv portion in the CAR vector was derived from the amino acid sequence of the second generation of CAR specific for MSLN or CEA, respectively, which was kindly provided by Prof. Hanmei Xu of China Pharmaceutical University, China. The sequences of other gene elements were obtained from the National Center for Biotechnology Information. The corresponding DNA sequence was synthesized by GENEWIZ® after codon optimization and then ligated into the pLV-puro vector via *Xho*Iand *Xba*I.

### Lentiviral engineering of T cells and target cells

Twenty-four hours prior to viral transduction, isolated T subtype cells were activated with human CD3/CD28 beads (Life Technologies) at a 1:3 cell to bead ratio in human T cell medium as described above. Activated T cells were transduced with the engineered virus in the presence of 1 μg/mL protamine sulfate (Sigma) by spinfection (800×*g*, 60 min) and were incubated overnight with the virus at 37 °C, 5% CO_2_ until the medium was replaced with fresh medium. Five days after transduction, modified T cells were harvested and CAR expression was detected by flow cytometry and western blot analyses.

All tumor cells, including AsPC-1, PANC-1, HT29, and U87, were cultured 36 h in advance to ensure that target cells would be in log phase at the time of lentivirus transduction. In a six-well plate, the fresh complete medium containing 6 μg/mL polybrene and the appropriate amount of virus were added to each well. After a 24-h incubation, the transduction medium was replaced with fresh complete medium to remove lentivirus and polybrene. Five days after transduction, transduced cells expressing RFP were selected with medium containing appropriate concentration of puromycin. Expression of transgenes in transduced tumor cells was confirmed by detecting fluorescent reporter proteins with flow cytometry and western blot analyses.

### Flow cytometry and western blot assays

For the flow cytometry assays, all cells were harvested and washed three times with FACS wash buffer (1 × PBS containing 0.5% BSA and 0.03% sodium azide) prior to flow cytometry. Surface staining of cells was performed with monoclonal antibodies directed against CD4, CD8, and CEA, which were purchased from Becton Dickinson (BD), while the monoclonal antibody against MSLN was purchased from R&D Systems. Transgenic populations with GFP expression for T cells or with RFP expression for tumor cells were analyzed on the fluorescein isothiocyanate (FITC) channel or the PerCP channel, respectively. For T cell activation, after overnight incubation, the T cell surface markers, CD25 and CD69, were detected using the APC-conjugated CD25 antibody (Biolegend) and APC-conjugated CD69 antibody (Biolegend), respectively. After a 30-min incubation period at 4 °C, protected from light, all cells were washed and analyzed by flow cytometry.

For western blot analysis, the proteins were extracted from modified cells and stored at − 80 °C. Western blots were performed using GFP primary antibody (abcam) and RFP primary antibodies (abcam).

### Enzyme-linked immunosorbent assays

For in vitro trials, primary T cells expressing CARs were mixed with various target cells at a 2:1 T cell to target cell ratio in a U-bottom 96-well plate. After overnight incubation, supernatants were harvested and assayed for the released IL-2, IFN-γ, TNFα, IL-4, IL-13, and IL-15 by ELISA assays, following manufacturer’s instruction (MultiSciences).

For in vivo trials, 100 μL of peripheral blood was collected from the treated mice at the designed time points and cytokines in blood serum, such as IL-2, IFNγ, TNFα, and IL-6, were analyzed by ELISA assays, according to the manufacturer’s instruction (MultiSciences).

### Quantitation of T cell proliferation

For proliferation assays, primary CD4^+^ T cells expressing the CARs or control CD4^+^ T cells were washed with PBS and then labeled with CellTrace Violet Kit (Life Technologies) at the final concentration of 5 μM, according to the manufacturer’s instructions. Cognate target cells (AsPC-1) expressing desired antigens and non-cognate target cells (HT29, U87, or PANC-1) were treated with 25 μg/mL mitomycin C (MedChem Express), resulting in target cells with a replication-incompetent state. Then, labeled T cells were co-cultured with treated target cells at a 2:1 effector/target ratio in human T cell medium and mixtured cells were collected for flow cytometry analysis. Lastly, the proliferation of CAR-T cells was assayed by monitoring the CellTrace Violet dilution after incubation for 3 days.

### Cytotoxicity assays

Primary CD8^+^ T cells endowing with CARs were co-cultured with target cells at a 2:1 effector/target ratio in 100 μL of T cell culture media supplemented with 10% FBS for 24 h at 37 °C. Cytolytic activity of modified T cells was determined by the level of lactate dehydrogenase (LDH) using Lactate Dehydrogenase Cytotoxicity Assay Kit (Cayman). The experimental groups and the control groups were designed according to the manufacturer’s protocol. Determination of LDH activity present in the sample was performed by the absorbance at 490 nm for each sample using a Multiskan FC plate reader (Thermo Scientific). Finally, T cell cytotoxicity was calculated by the following formula: specific cytotoxicity (%) = (mixture cell experiment − effector cell spontaneous − target cell spontaneous − medium control)/(target cell maximum − target cell spontaneous − medium control) × 100.

### Xenograft mouse models and living imaging assays

Female nude mice, 7–9 weeks of age, were housed in the Laboratory Animal Research Center of China Pharmaceutical University (CPU), and all protocols were performed as approved by the CPU Institutional Animal Care and Use Committee. To carry out the xenograft models, nude mice were randomly divided into six groups (*n* = 5) and were intravenously inoculated with 5 × 10^5^ engineered tumor cells per mouse (day 0), including AsPC-1 cells, HT29 cells, U87 cells, or PANC-1 cells. After 7 days, 2 × 10^6^ CAR-T cells or wild T cells per mouse were infused intravenously.

To characterize the tumor killing effect in vivo, tumor burden of each mouse was measured using the In-Vivo Imaging System Fx Pro (Carestream Health) following the instrument’s instructions at 7 days, 14 days, 21 days, 28 days, and 35 days after tumor injection. In this experiment, the cytotoxicity of modified T cells was calculated according to the radiance intensity in the region of the tumor site. Finally, a ratio of the mean fluorescence intensity (MFI) of tumor cells in the mice treated with engineered T cells to that of the mice treated with wild T cells (No CAR T) was calculated to enumerate redirected cytotoxic activities of engineered T cells at 35 days after tumor injection.

### Quantitation of T cell counts and cytokine production in vivo

Furthermore, 80 μL of peripheral blood of each nude mouse was drawn at 1 days, 7 days, 14 days, 21 days, and 28 days after modified T cell infusion (totally five times) to determine T cell expansion and persistence in the blood at the stated time points by flow cytometry detection of GFP expression. In this experiment, a ratio of the number of T cells at the indicated time in the mice treated with effector cells to T cell counts at 24 h after modified T cell infusion was calculated for each sample to enumerate the relative survival of injected T cells.

At the end of the experiment, serum cytokine concentrations were monitored in peripheral blood of each nude mouse using the Elisa Kit, including IL-2, IFNγ, IL-6, and TNFα.

### Statistical analysis

Statistical significance were calculated using GraphPad software, version 6.0. Unless otherwise stated, in vitro and in vivo data were analyzed using the two-tailed Student’s *t* test. Data acquired from in vitro assays using experimental replicates (*n* = 3) and other data acquired from in vivo assays using biological replicates (*n* = 5) are presented as mean ± SD. All graphs were also generated using GraphPad prism. A *p* value less than 0.05 was considered statistically significant. Significance of findings was defined as n.s. or not significant *p* > 0.05, *p < 0.05, **p < 0.01, and ***p < 0.001. For Figs. 2c and 3c, e, f, statistical significance was calculated using “experiment group” vs “No CAR T cell treatment group.” For Fig. 2g and Additional file 1: Figure S4, statistical significance was calculated using “experiment group” vs “CEA-CAR T cell treatment group.” For Additional file 1: Figure S3, statistical significance was calculated using “experiment group” vs “dCAR T cell incubated with AsPC-1 cell group.”

## Results

### Construction of effector cells and target cells

**Fig. 1:**
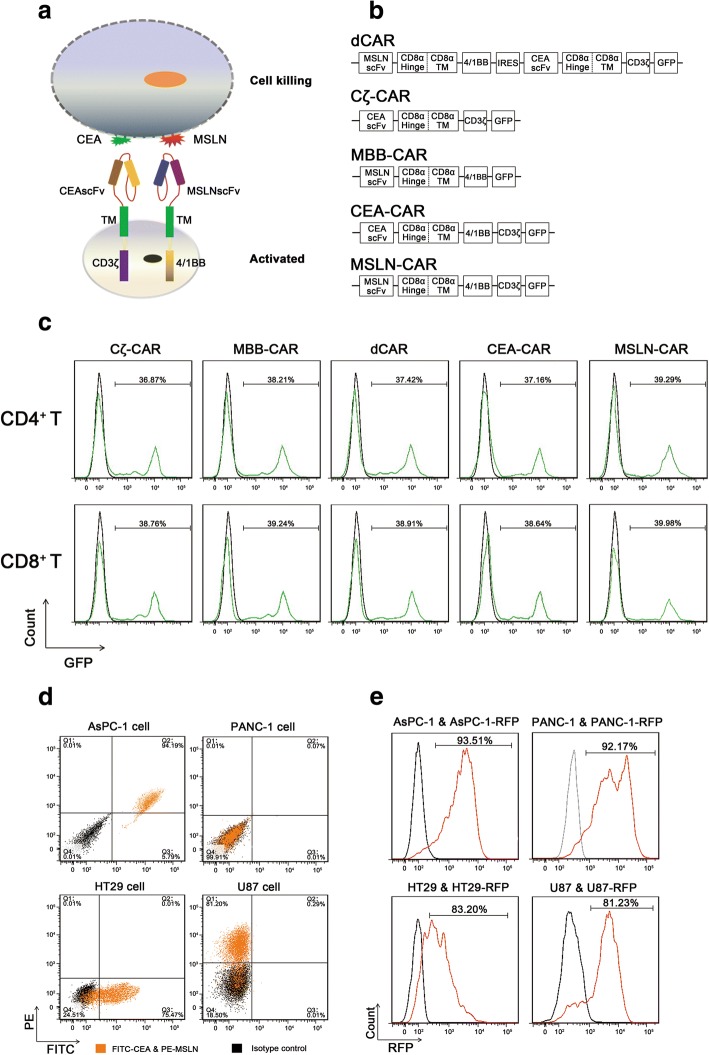
Construction of effector cells and target cells. **a** Schematic representation of dCAR-engineered T cells. Engineered T cells endowed with the dCAR structure are activated in the presence of CEA and MSLN and eliminate target cells. **b** Structure of the plasmids used to construct modified T cells. After lentivirus transfection, effector cells yield expression of the novel dCAR structure consisting of anti-MSLN scFv, cytoplasmic domain of 4/1BB, anti-CEA scFv, cytoplasmic domain of CD3ζ, and green fluorescence protein (GFP) sequences. Simultaneously, we also constructed two negative controls, Cζ-CAR and MBB-CAR, and two positive controls, CEA-CAR and MSLN-CAR. **c** CAR-engineered T cells were successfully constructed by lentivirus transfection. With endogenous GFP expression, we measured the transduction efficiency by flow cytometry assays, quantifying fractions of CAR positive-CD4^+^ T and CAR positive-CD8^+^ T cells in different CAR groups. **d** Antigen expression on the cognate and non-cognate tumor cells. By flow cytometry assays, AsPC-1 cells highly expressed both CEA and MSLN while PANC-1 cells did not express the above antigens. In addition, HT29 cells could only highly express CEA and U87 cells only express MSLN. **e** Detection of the transfection efficacy of modified tumor cells. After puromycin screening, the transduction efficiencies were determined to be 93.51%, 92.17%, 83.20%, and 81.23% by flow cytometry assays for AsPC-1-RFP cells, PANC-1-RFP cells, HT29-RFP, and U87-RFP, respectively

To demonstrate that both T cell activation and co-stimulation signals can be supplied by two distinct receptors, we have constructed a novel CAR model, termed dual-receptor CAR (dCAR), according to the dual-signal pathway of natural T cells. Briefly, in this CAR, a CD3ζ-mediated activation signal was dependent upon recognition of the tumor antigen CEA and a 4/1BB co-stimulation signal domain was subjected to the second receptor specific for tumor antigen MSLN (Fig. 1a). Insertion of a GFP tag would facilitate detection of dCAR expression present on modified T cells. We also provided two negative controls, single-receptor CARs including CEA-CD3ζ CAR (Cζ-CAR) and MSLN-4/1BB CAR (MBB-CAR), and two positive controls, the second-generation CAR specific for CEA (CEA-CAR) and the second-generation CAR specific for MSLN (MSLN-CAR) (Fig. 1b, Additional file 1: Figure S1). After lentivirus transfection, modified T cells typically yielded expression of antigen receptors in 36–40% of T cells by detection of GFP expression with flow cytometry assays and WB assays (Fig. 1c, Additional file 1: Figure S2a). In all subsequent experiments, six groups of T cells, endowing with Cζ-CAR, MBB-CAR, dCAR, CEA-CAR, MSLN-CAR, or neither (mock), were analyzed and used to verify the effectiveness and safety of dCAR-T cell therapy.

In this study, we selected the pancreatic cancer cell line AsPC-1 as a target cell due to its high expression of both CEA and MSLN (Fig. 1d). In order to facilitate the detection of target cells in in vitro and in vivo trials, target cells highly expressed the RFP via lentivirus transfection. In addition, we also constructed some non-cognate target cells with expression of RFP, such as, HT29 cells expressing CEA yet lack of MSLN expression, U87 cells expressing MSLN yet lack of CEA expression, and PANC-1 cells without expression of CEA or MSLN (Fig. 1d). After screening with the puromycin-containing medium, the transduction efficiency of RFP-modified-AsPC-1 cells, RFP-modified-HT29 cells, and RFP-modified-U87 cells, RFP-modified-PANC-1 cells was 93.51%, 83.20%, 81.23% and 92.17%, respectively (Fig. 1e). The data obtained from WB assays were consistent with that of the flow cytometry assays, indicating that cognate and non-cognate target cells have been successfully constructed (Additional file 1: Figure S2b).

### Determination of the effector/target ratio

**Fig. 2:**
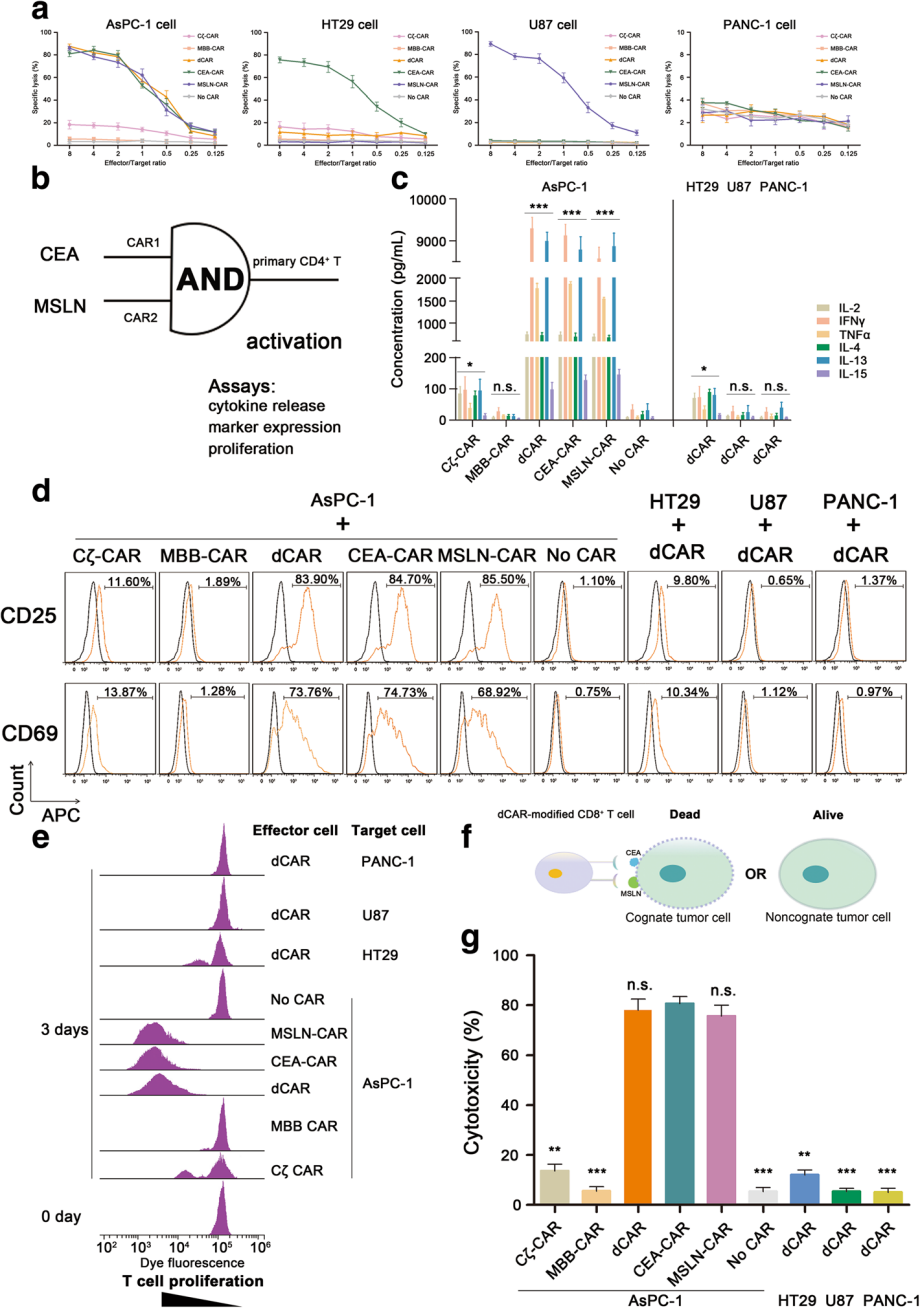
Combinatorial antigen requirement for T cell activity in vitro. **a** Modified T cells or wild T cells were incubated at indicated with various tumor cells at an effector/target rations of 8:1, 4:1, 2:1, 1:1, 1:2, 1:4, or 1:8. After a 24-h incubation, target cell lysis was measured by LDH release in the supernatant. The optimal effector/target ratio in this research was determined to be 2:1. In addition, dCAR-T cells could specifically lyse AsPC-1 cells yet do not eliminate HT29 cells, U87 cells, and PANC-1 cells (*n* = 3, error bars denote standard deviation). **b** Activation of dCAR-engineered CD4^+^ T cells required cognate target cells. The primary CD4^+^ T cells were modified with dCARs by lentivirus transfection, and cell activation assays were performed with an “AND logic gate” strategy, including cytokines release, marker expression, and T cell proliferation. **c** Released cytokines in each sample were quantified by enzyme-linked immunosorbent assay, including IL-2, IFNγ, TNFα, IL-4, IL-13, and IL-15. All cytokines were significantly produced when dCAR-T cells were exposed to AsPC-1 cells yet not when exposed to non-cognate tumor cells (HT29 cells, U87 cells, or PANC-1 cells). For conventional CAR-T (CEA-CAR or MSLN-CAR) cell treatment, similar cytokines were obtained (*n* = 3, error bars denote standard deviation). **d** Monitoring T cell activation by CD25 and CD69 expression. T cell activation marker, CD25 or CD69, was significantly expressed on dCAR-T cells or conventional CAR-T cells compared with that of other single-receptor modified T cells in the presence of AsPC-1 cells (*n* = 3). **e** Combinatorial antigen-dependent T cell proliferation. Data showed that dCAR-T cells have a high proliferation activity in the presence of cognate tumor cells expressing CEA and MSLN, which was similar to that of conventional CAR-T cells against target cells. Interestingly, Cζ-CAR-modified T cells showed a lower proliferation capacity, indicating that the CD3ζ signaling pathway is not sufficient to trigger T cell activation (*n* = 3). **f** dCAR-engineered CD8^+^ T cells yield specific target cell killing in vitro. **g** Cytotoxicity mediated by dCAR-CD8^+^ T cells in a 24-h experiment. After an overnight incubation, significant cytotoxicity was observed in dCAR-T cells co-cultured with AsPC-1 cells, approximately 85% of target cell apoptosis (*n* = 3, error bars denote standard deviation)

Cytotoxicity against cognate target cells expressing both CEA and MSLN was, as expected, endowed by dCAR expression. To minimize the effect of differential CAR expression, CAR-T cells after each transfection were measured by flow cytometry assays prior to in vitro and in vivo efficacy studies. To effectively characterize the CAR-modified T cell activity, we first performed a 24-h cytotoxicity assay at varying effector-to-target (E:T) cell ratios. In these experiments, the cytotoxicity of modified T cells was measured by detection of the level of lactic dehydrogenase (LDH) release from the apoptotic target cells in the supernatant. Our data showed that dCAR cells, CEA-CAR T cells and MSLN-CAR T cells specifically lysed 81–87% cognate target cells at the 2:1 effector/target ratio, whereas Cζ-CAR T cells had approximately 18% cytotoxicity yet MBB-CAR T cells did not lyse cognate target cells (Fig. 2a). Therefore, the optimal effector-to-target ratio in this research was determined to be 2:1, which was used in subsequent experiments.

In addition, for the CEA-expressing tumor cell line, HT29 cells, CEA-CAR T cells could achieve significant target cell killing (approximately 70%), whereas other modified effector cells have no ability to eliminate target cells. For U87 cells, which have MSLN expression yet not CEA expression, results showed that only MSLN-CAR T cells could achieve remarkable target cell killing. For PANC-1 cells that are lack of both CEA and MSLN, data showed that all CAR-modified T cells and wild T cells did not exert significant cytotoxicity. In summary, the novel dual-targeting modified T cells, dCAR-T cells, yielded significant anti-tumor activity only when two antigen receptors were simultaneously targeted; however, dCAR-T cells cannot cause the target cell apoptosis when only the single pathway *(*e.g., Cζ or MBB) was activated. dCAR-T cells achieved significant cytotoxicity only in the presence of AsPC-1 cells expressing both CEA and MSLN, which rivaled that obtained with conventional CAR-T cells, CEA-CAR T cells, or MSLN-CAR T cells. Therefore, CAR-T cells endowed with a dual-targeting receptor have significant specificity and safe activity, mitigating “on-target, off-tumor” toxicity derived from conventional CAR-T cell therapy.

### Combinatorial antigen requirement for T cell activation

We next tested the activation of dCAR in modified T cells exposed to target cells, including AsPC-1 cells and PANC-1 cells. All of the tested CD4^+^ T cell responses, including cytokine production (such as IL-2, IFNγ, TNFα, IL-4, IL-13, and IL-15) and expression of activation markers on T cell surface (such as CD69 and CD25), showed the requirement for T cell activation with the two distinct antigens (Fig. 2b).

Production of various cytokines was maximal when dCAR-T cells were co-cultured with AsPC-1 cells expressing both CEA and MSLN, compared with that of Cζ-CAR or MBB-CAR-modified T cells stimulated by AsPC-1 cells. In addition, cytokine production of activated dCAR-T cells was comparable with that obtained with modified T cells expressing the conventional CAR (CEA-CAR or MSLN-CAR) (Fig. 2c). Briefly, during the experiment, dCAR-T cells could release significant levels of cytokines only in the presence of cognate tumor cells expressing both CEA and MSLN, including 751 pg/mL IL-2, 9297 pg/mL IFNγ, 1777 pg/mL TNFα, 732 pg/mL IL-4, 8991 pg/mL IL-13, and 99 pg/mL IL-15. For the single-receptor CARs, Cζ-CAR and MBB-CAR, cognate tumor cells stimulate modified T cells to release low level of cytokines, which is similar with that of wild T cells (No CAR).

In addition, T cells could significantly express the activation markers, including CD69 and CD25, within hours after cell activation. In this study, as was observed with the conventional CAR-T cells, T cells expressing the dCAR displayed a high CD69 and CD25 expression pattern upon activation compared with single-receptor modified T cells (Fig. 2d). Data showed that the modified T cells required an activated dual-signal pathways, including CD3ζ and 4/1BB, for activation in the presence of cognate tumor cells.

In summary, the novel mode of dCAR renders T cells with significant activity through targeting the distinct tumor-associated antigens, improving the specificity and safety of CAR-modified T cells against tumor cells.

### Combinatorial antigen control over T cell proliferation

Cognate tumor cell-induced proliferation of CAR-T cells is an indispensable prerequisite for amplification of T cell therapeutic activities; however, uncontrolled T cell proliferation leads to some severe toxicities. We next examined whether the proliferation of dCAR-T cells was dependent upon the cognate tumor cells expressing both CEA and MSLN.

Modified CD4^+^ T cells were labeled with an intracellular fluorescent dye to monitor T cell proliferation and then were co-cultured with replication-incompetent target cells. In a flow cytometry experiment, data showed that dCAR-T cells had a high proliferation activity in the presence of cognate tumor cells expressing CEA and MSLN, which was similar to that of conventional CAR-T cells (CEA-CAR T or MSLN-CAR T) (Fig. 2e). On the contrary, MBB-CAR-modified T cells or wild T cells had no significant cell proliferation in the presence of cognate target cells, while Cζ-CAR-modified T cells showed a lower proliferation capacity, indicating that the CD3ζ signaling pathway is vital for the T cell activation yet by itself is not sufficient to completely initiate T cell activity. Additionally, data showed that dCAR-T cells incubated with non-cognate tumor cells (HT29 cells, U87 cells, and PANC-1 cells) had no remarkable proliferation.

In summary, proliferation of dCAR-T cells indeed required two distinct tumor antigens, whereas only one antigen did not thoroughly induce dCAR-T cell proliferation. Therefore, controlling the degree of modified T cell expansion by targeting the two tumor-associated antigens may be an effective approach to control the strength of the immune response, mitigating the serious “on-target, off-tumor” toxicity.

### Combinatorial antigen control over tumor killing in vitro

Next, we evaluated the ability of two distinct tumor-associated antigens (i.e., CEA and MSLN) to redirect dCAR-T cells to cognate tumor cell AsPC-1, using antigen-negative PANC-1 cell line as a control. In general, a major purpose of CARs is to redirect human CD8^+^ T cell to selectively recognize tumor cells expressing antigens of interest, thereby improving therapeutic accuracy and tumor elimination. Thus, we examined whether primary human CD8^+^ T cells expressing the dCAR could mount a cytotoxic response that was still antigen specific but gated by the two tumor-associated antigens. The cytotoxic activity of CD8^+^ T cells expressing the dCAR was then quantified based on the level of LDH released from apoptotic target cells in the supernatant (Fig. 2f).

After an overnight incubation, significant cell-mediated cytotoxicity was observed in dCAR-T cells co-cultured with cognate tumor cells expressing both CEA and MSLN, with approximately 80% of target cell apoptosis which was similar to that of conventional CAR-T cells (CEA-CAR T or MSLN-CAR T) (Fig. 2g). Efficient killing of the cognate target cells was not observed when single-receptor CAR-T cells (i.e., Cζ-CAR-T cells and MBB-CAR-T cells) or wild T cells were added. In addition, killing of non-cognate target cells (HT29, U87, or PANC-1) was not observed in co-culture with dCAR-modified T cells, confirming that dCAR-T cell have a specific activity.

In summary, the results with primary human CD8^+^ T cells confirmed that dCAR-T cells showed highly potent cytotoxicity for cognate tumor cells expressing two distinct tumor-associated antigens, suggesting that complete tumor eradication had been achieved. However, based on our data, we envision that the single antigen-expressing tumor do not initiate dCAR-T cell with significant activity, providing a precise cell-based therapeutic approach in a dual-targeting combinatorial antigen manner (Additional file 1: Figure S3). In addition, dCAR-modified CD4^+^ T cells also have cytotoxicity against cognate tumor cells (approximately 30%); therefore, CD4^+^ T cells possess lower target cell killing capacity compared with CD8^+^ T cells (Additional file 1: Figure S4).

### dCAR-engineered T cells on tumor clearance in vivo

**Fig. 3:**
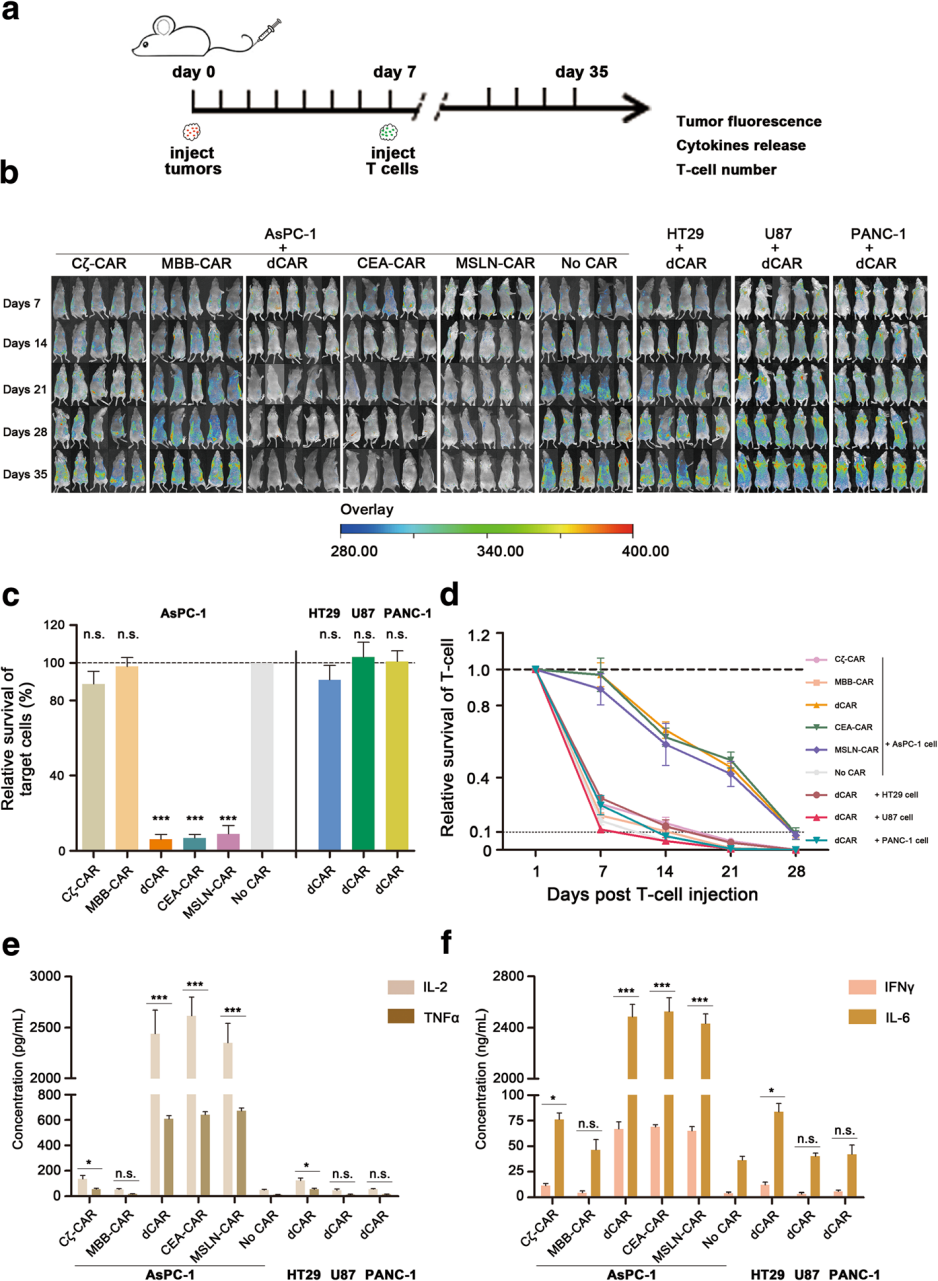
dCAR-engineered T cells on tumor clearance in vivo. To investigate the activity of dCAR-T cells in vivo, we evaluated the pancreatic cancer regression in immunodeficient mice. **a** Schematic of the mouse treatment strategy. Day 0, all tumor cells, including AsPC-1 cells, HT29 cells, U87 cells, or PANC-1 cells, were injected into nude mice intravenously (*i.v.*). Mice were treated with various effector cells at 7 days after tumor injection, and then the survival of target cells, cytokine release, and T cell number were detected. **b** After effector cell injection, the tumor regression was followed by in vivo living image of the mean fluorescence intensity from RFP-engineered tumor cells (*n* = 5). **c** Cytotoxic activities of engineered T cells were characterized based on the mock T cell cytotoxicity at 35 days after tumor injection. Data showed that the relative cytotoxic activity of effector cells is approximately 93.8%, having a significant difference relative to that of mock T cells (*n* = 5, error bars denote standard deviation). **d** The proliferation of modified T cells was measured in vivo. Data showed that dCAR-T cells provided significantly greater proliferation compared with the single-receptor structure, including Cζ-CAR and MBB-CAR (*n* = 5, error bars denote standard deviation). **e**, **f** Effector cells released various cytokines, including IL-2, TNFα, IL-6, and IFNγ. In our experiment, significant cytokine production had achieved in mice treated with dCAR-T cells, which was similar to that of conventional CAR-T cells (CEA-CAR T or MSLN-CAR T) (*n* = 5, error bars denote standard deviation)

To investigate the activity of dCAR-T cells in vivo, we evaluated tumor regression in a xenograft model using RFP-modified tumor cells. In this model, mice were treated with various effector cells at 7 days after tumor injection (Fig. 3a). Briefly, to demonstrate the efficacy and safe of the novel structure of dual-receptor CAR in vivo, mice were inoculated with various cancer cells, including AsPC-1 (MSLN^+^ CEA^+^), HT29 (MSLN^−^ CEA^+^), U87 (MSLN^+^ CEA^−^), or PANC-1 (MSLN^−^ CEA^−^), followed by intravenous injection of modified T cells or mock T cells 7 days later. The tumor regression was followed by in vivo living image of the mean fluorescence intensity from RFP-engineered tumor cells. Based on our in vivo results, 28 days after T cell infusion, mice that received dCAR-T cells showed a marked reduction in tumor burden and even complete tumor remission, which was similar to that of the conventional CAR (CEA-CAR or MSLN-CAR) T cell treatment (Fig. 3b). However, for cognate tumor cells (AsPC-1), the tumor burden in mice treated with MBB-CAR T cells or mock T cells gradually increased. In addition, mice injected Cζ-CAR T cells did not show a significant decrease in tumor burden, suggesting that the tumor eradication had not been achieved. Meanwhile, mice bearing non-cognate tumor cells (HT29, U87, or PANC-1) had underwent substantial increase in tumor burden even after dCAR-T cell infusion.

Next, the cytotoxic activities of engineered T cells were characterized based on the mock T cell cytotoxicity at 35 days after tumor injection. In mice that were injected with AsPC-1 cells followed by dCAR-T cells, the relative cytotoxic activity of effector cells is approximately 93.8%, having a significant differences relative to that of mock T cells (*p* < 0.001) (Fig. 3c). Similar results were obtained in mice treated with conventional CAR T cells, such as CEA-CAR T cells (93.2%) or MSLN-CAR T cells (91%). Taken together, in agreement with our in vitro results, dCAR-T cells can eliminate cognate tumor cells with comparable efficacy to conventional CAR-T cells in vivo; however, other mice treated with Cζ-CAR cells or MBB-CAR cells failed in inhibiting the cognate tumor cell growth.

### T cell proliferation and cytokine production in vivo

We next investigated modified T cell proliferation during target cell elimination in mice. After effector cell infusion, increased T cell counts in the peripheral blood were detected at 24 h and every other 7 days for 4 weeks during tumor burden decrease. Results revealed that dCAR-T cells provided significantly greater proliferation compared with Cζ-CAR T cells or MBB-CAR T cells (Fig. 3d).

In general, effector cells rejected the tumor cells, accompanying with cytokine release, including IL-2, TNFα, IL-6, and IFNγ. In our experiment, production of various cytokines had achieved in mice infused with the dCAR or conventional CAR-T cells (Fig. 3e, f). Briefly, dCAR-T cells in mice bearing the cognate tumor cells (AsPC-1) released high levels of cytokines, including 2320 pg/mL IL-2, 609 pg/mL TNFα, 2486 ng/mL IL-6, and 67 ng/mL IFNγ, which was similar to that observed for cytotoxicity. The significant cytokine production had also been observed in response to CEA-CAR T cells or MSLN-CAR T cells. Furthermore, other engineered T cells did not produce significant levels of cytokines.

Collectively, the above experiments demonstrated that the activity of dCAR-T cells requires both the first signal pathway (antigen recognition signaling domain, CD3ζ) and the second signal pathway (co-stimulation signaling domain, 4/1BB). Based on the data, dCAR-T cell cytotoxicity is comparable to that of conventional CAR-T cells. For the single-receptor CAR bearing one signal pathway in this research, including Cζ-CAR and MBB-CAR, effector cells had no significant activation activity and did not exert remarkable cytotoxicity even in the presence of cognate tumor cells (AsPC-1) expressing both MSLN and CEA. Therefore, the dual signaling pathway is a prerequisite for T cell activity, whereas the CAR containing a single-signaling pathway endows T cells with resting state, including activation, proliferation, and cytotoxicity. Excitingly, a novel type of CAR-T cells, termed dCAR-T, was designed with high specificity, that is, significant cytotoxicity for tumor cells that express both distinct antigens yet no cytotoxicity for single antigen-positive tumor cells. Finally, this novel CAR model has a good safety and high activity, alleviating “on-target, off-tumor” toxicity in the presence of the known tumor-associated antigens.

## Discussion

Chimeric antigen receptor modified T cells have achieved promising clinical responses in the treatment of the hematological malignancies in recent years [28, 29]. With advance in tumor microenvironment, some approaches have been developed to enhance the efficacy of CAR-T cells against various solid tumors, resulting in some ongoing clinical trials [30]. While achieving significant therapeutic effects with the immune cell therapy, patients are often accompanied with severe side effects, especially “on-target, off-tumor” toxicity [16, 18]. The antigens used for CAR structure are often tumor-associated antigens co-expressed both on cancerous tissues and adjacent tissues; therefore, conventional CAR-T cells effectively rejected the tumor cells while damaging the normal tissues expressing the targeted antigen. In general, the two signaling domains derived from the intracellular part of T cell receptors are connected in a tandem manner and controlled by a targeted-antigen receptor; therefore, one antigen can control the two signaling domains, which increases the chance of damaging normal cells expressing interested antigens specific for CAR structure present on T cells [31].

**Fig. 4:**
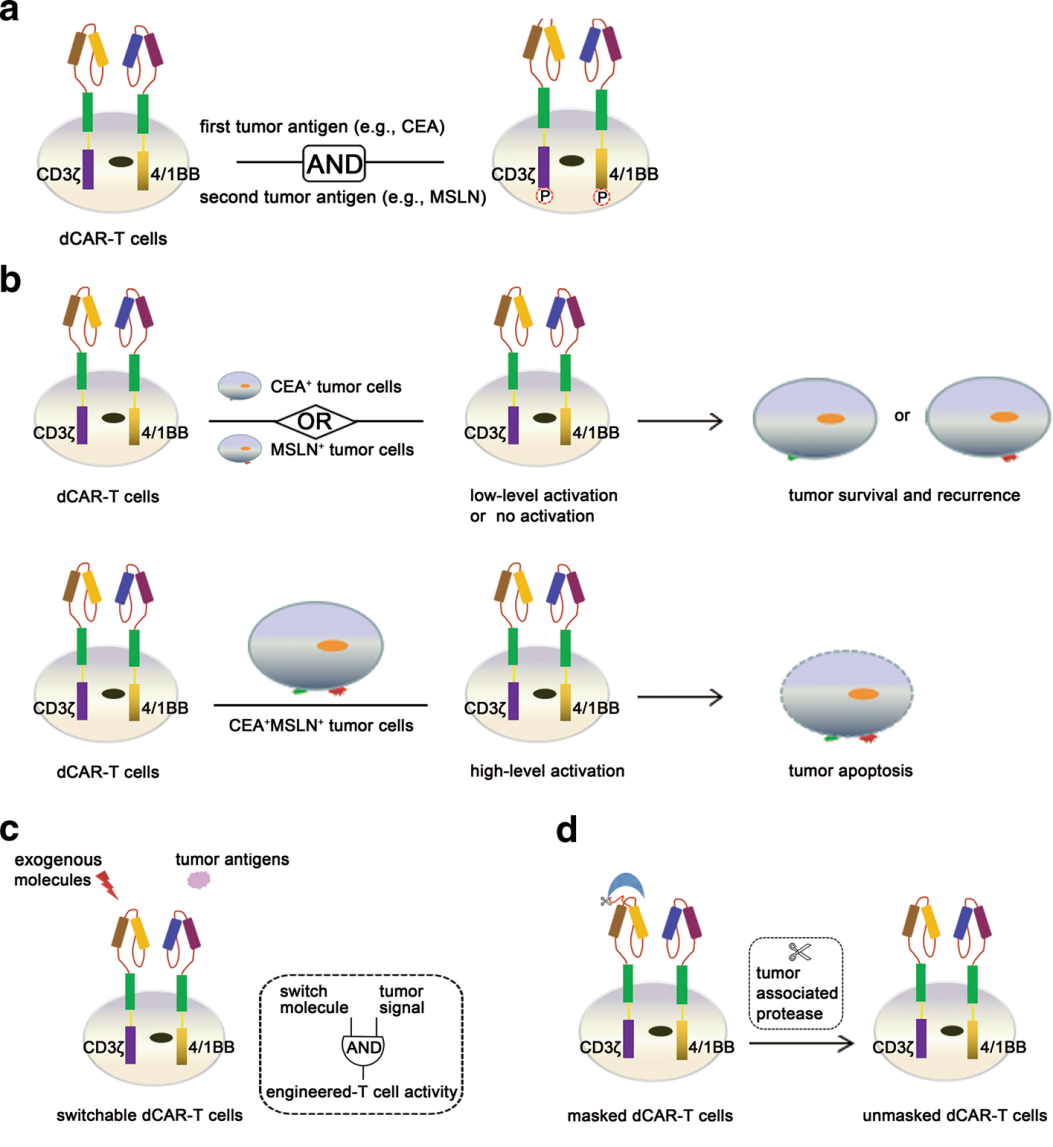
General strategies that improve the therapeutic safety of engineering dCAR-T cells. **a** In the presence of the first tumor antigen (e.g., CEA) >and the second tumor antigen (e.g., MSLN), the intracellular CD3ζ and 4/1BB signal domain were phosphorylated, resulting in the fact that cognate tumor cells expressing MSLN and CEA are required for dCAR-T cell activity. **b** For tumor cells expressing a single tumor-associated antigen, dCAR-T cells have low-level activation in the presence of CEA yet no activation in the presence of MSLN, resulting in tumor survival and recurrence. For dual antigen-expressing tumor cells, dCAR-T cells have high-level activation and exert significant cytotoxicity, resulting in tumor apoptosis. **c** To achieve the accurate control of dCAR-T cells in vitro and in vivo, the CD3ζ signaling pathway of dCAR structure was regulated by an exogenous molecular. Therefore, switchable dCAR-T cells eliminate cognate tumor cells expressing the tumor antigens specific for the second CAR of this model only in the presence of the switch molecule. **d** To decrease normal tissue damage, a cleavable masking peptide could be re-engineered into the N-terminal of CEA-CD3ζ pathway domain, thereby blocking the antigen recognition domain of the CEA scFv. Thus, masked dCAR-T cells eradicate cognate tumor cells only in the presence of the tumor-associated protease locally active in tumor environment, thus enabling dCAR-T cell to differentiate tumor tissue and normal tissue

To achieve safe cytotoxicity of CAR-T cells, several strategies have been described that use a novel CAR structure or a tumor-specific antigen to control the activation of T cell receptor. In the present study, the activity of CAR-engineered T cells was significantly improved through the action of a dual-receptor CAR paradigm. For the first time, we designed a dual-receptor CAR model that contains two physically separate structures, CEA-CD3ζ and MSLN-4/1BB signaling domains specific for CEA and MSLN, respectively (Fig. 4a). The dCAR-engineered T cell cytotoxicity was effectively controlled with cognate tumor cells (AsPC-1) expressing CEA and MSLN, thereby mitigating “on-target, off-tumor” toxicity and improving the safety of T cell therapy. In addition, dCAR-T cells cleared cognate tumor cells with a similar cytotoxicity comparable to that of conventional CAR-T cells (CEA-CAR T or MSLN-CAR T) in in vitro and in vivo experiments, thereby promoting their applications.

Based on our results, cognate tumor cells expressing MSLN and CEA are required for dCAR-T cell activity, including activation, proliferation, and cytotoxicity. Engineered T cells designed here are not activated when a single-receptor of dCAR-T cells interacts with the corresponding antigen, which triggers phosphorylation of the intracellular 4/1BB signal domain yet is not able to initiate T cell activation because the first signaling pathway (CD3ζ) is vital for the activity of T cells. Hence, dCAR-engineered T cells have no any cytotoxicity for normal tissues expressing only the tumor-associated antigen MSLN. Similar with the first generation of CAR-T cells, dCAR-T cells designed here have low-level activation in the presence of CEA antigen, resulting in lower cytotoxicity against target cells. However, the activation of dCAR-T cells targeted by CEA is not sufficient to achieve significant cytotoxicity (Fig. 4b). Theoretically, targeting the CEA receptor expressed on T cell surfaces triggers phosphorylation of the intracellular CD3ζ signal domain and then initiates T cell activation with slight and transient characteristics. Therefore, the novel dCAR-T cells can have slight damage for normal tissues expressing only the antigen CEA, or even no damage. Our experiments showed that the modified T cell activity derived from this model where only the CEA-CD3ζ signaling pathway was activated is very limited, resulting from the fact that the intracellular phosphorylation may fail to exert the persistent T cell activity. Therefore, the co-stimulatory signaling pathway (MSLN-4/1BB) in the novel dCAR is also crucial for the activity of engineered T cells. As expected, dCAR-T cells can exert strong cytotoxicity for dual antigen-expressing tumor cells (AsPC-1), which is comparable with that of conventional CAR-T cells. The synergistical phosphorylation of the CD3ζ and 4/1BB signal domains in the presence of cognate tumor cells expressing CEA and MSLN ultimately rendered T cells with complete activation and significant cytotoxicity. Thus, the dCAR-T cell activity was strictly dependent on cognate tumor cells, mitigating the “on-target, off-tumor” toxicity.

In fact, engineered T cells bearing the activated CD3ζ signaling pathway have a potential to exert cytotoxicity for CEA single-positive target cells. To further improve the safety of dCAR-T cell therapy, we envision that the CD3ζ signaling pathway of dCAR structure could be characterized to a novel model that can be precisely regulated. With the advance of a CAR diagram and a switch molecule, the dCAR model could be regulated via the first signaling pathway (CD3ζ) in the presence or absence of the exogenous factors, achieving the precise control of injected dCAR-T cells [32–34] (Fig. 4c). According to the characteristics of the solid tumor microenvironment, the new CAR structure, termed masked CAR, rendered the CD3ζ signaling pathway with an activated state only at the tumor site as a result of the proteases, thereby enabling dCAR-T cells to recognize the tumor tissue [35] (Fig. 4d).

In conclusion, because TAAs widely are expressed in many kinds of human solid carcinomas, this dCAR strategy offers a conception toward the design of CARs capable of targeting cancer cells lacking the tumor-specific antigens. Dual-targeted CAR-T cells can be precisely localized at the tumor site and can exert high cytotoxicity against tumor cells while the adjacent tissues are not damaged, enabling accurate application of CAR-T cell therapy.

## Conclusions

Based on our data, a novel type of CAR-T cells, dCAR-T cells, can exert significant cytotoxicity only in the presence of cognate tumor cells (AsPC-1) expressing both CEA and MSLN, which is comparable with that obtained with conventional CAR-T cells and alleviates “on-target, off-tumor” toxicity. In summary, the combination therapy using two distinct TAAs to regulate dCAR-T cell activity is becoming increasingly prospective in the field of cell-based cancer immunotherapy.

## Additional file

Additional file 1: **Figure S1.** Schematic representation of CAR-engineered T cells in this research. **Figure S2.** Detection of effector cells and target cells.** Figure S3.** dCAR-mediated activation and co-stimulation of CD8^+^ T cells facilitates significant cytotoxicity and specific activity. **Figure S4.** dCAR-engineered CD4^+^ T cells could yield slight cytotoxicity compared with CAR-modified CD8^+^ T cells. (DOCX 911 kb)

## Abbreviations

APC: Allophycocyanin
BSA: Bovine serum albumin
CARs: Chimeric antigen receptors
CEA: Carcino-embryonic antigen
dCAR: Dual-receptor chimeric antigen receptor
FBS: Fetal bovine serum
FITC: Fluorescein isothiocyanate
GFP: Green fluorescence protein
HER-2: Human epidermal growth factor receptor 2
IRES: Internal ribosome entry site
LDH: Lactate dehydrogenase
MFI: Mean fluorescence intensity
MSLN: Mesothelin
PBMCs: Peripheral blood mononuclear cells
PBS: Phosphate buffer saline
RFP: Red fluorescence protein
TAAs: Tumor-associated antigens
