# Supplementary material for: Recombination of a dual-CAR-modified T lymphocyte to accurately eliminate pancreatic malignancy
Source: J Hematol Oncol. 2018 Aug 13;11:102. doi: 10.1186/s13045-018-0646-9 (PMC6090669; doi:10.1186/s13045-018-0646-9)
Supplement: Supplementary file 1 — Figure S1. Schematic representation of CAR-engineered T cells in this research. Figure S2. Detection of effector cells and target cells. Figure S3. dCAR-mediated activation and co-stimulation of CD8+ T cells facilitates significant cytotoxicity and specific activity. Figure S4. dCAR-engineered CD4+ T cells could yield slight cytotoxicity compared with CAR-modified CD8+ T cells. (DOCX 911 kb) [file 13045_2018_646_MOESM1_ESM.docx]

**Additional file 1**

**Recombination of a dual-CAR modified-T lymphocyte to accurately eliminate pancreatic malignancy**

Erhao Zhang^1,2^, Peiwei Yang^1^, Jieyi Gu^1^, Heming Wu^3^, Xiaowei Chi^1^, Chen Liu^1^, Ying Wang^1^, Jianpeng Xue^1,4^, Weiyan Qi^1,4^, Qingbo Sun^1^, Shengnan Zhang^1^, Jialiang Hu^1,4,*^ and Hanmei Xu^1,4,5,*^

**Correspondence:** Jialiang Hu, E-mail: [haobo89@163.com;](mailto:jialiang_hu51@aliyun.com;) Hanmei Xu, E-mail: 13913925346@126.com

*Shared senior authorship

**
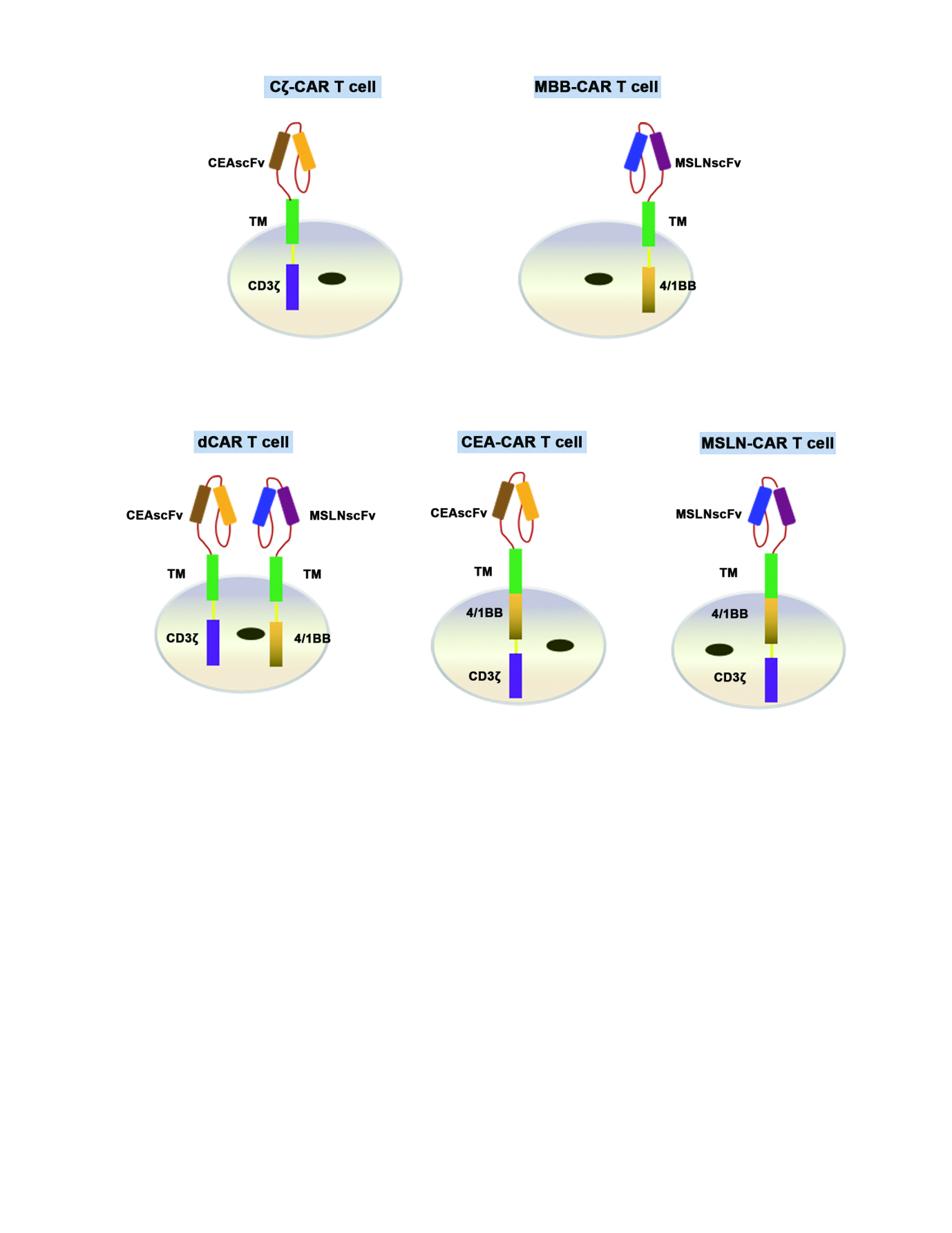
**

**Figure S1.** Schematic representation of CAR-engineered T cells in this research. To facilitate the verification of the dCAR-T cell activity, five groups of engineered-T cells, endowing with Cζ-CAR, MBB-CAR, dCAR, CEA-CAR, or MSLN-CAR, were constructed.

**
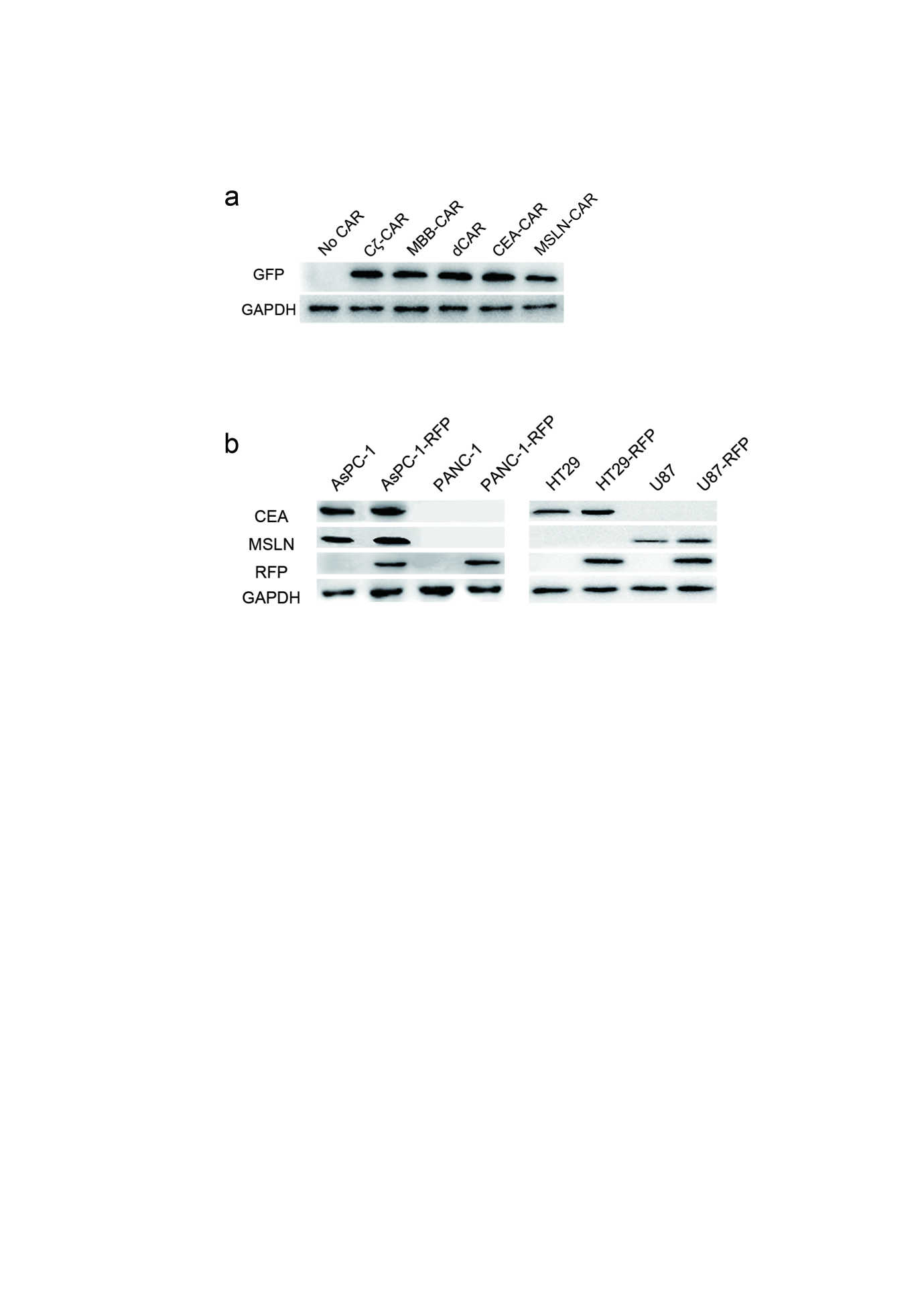
**

**Figure S2.** Detection of effector cells and target cells. **a** After lentivirus transfection, modified-T cells typically yield expression of antigen receptors by detection of GFP expression. With use of an anti-GFP antibody, the expressions of GFP on the various engineered-T cells were determined by WB assays. **b** In order to facilitate detection of target cells *in vitro* and *in vivo*, target cells were transfected to express RFP. WB assays showed that RFP-modified target cells, including AsPC-1-RFP cells, PANC-1-RFP cells, HT29-RFP cells and U87-RFP cells, have been successfully constructed.


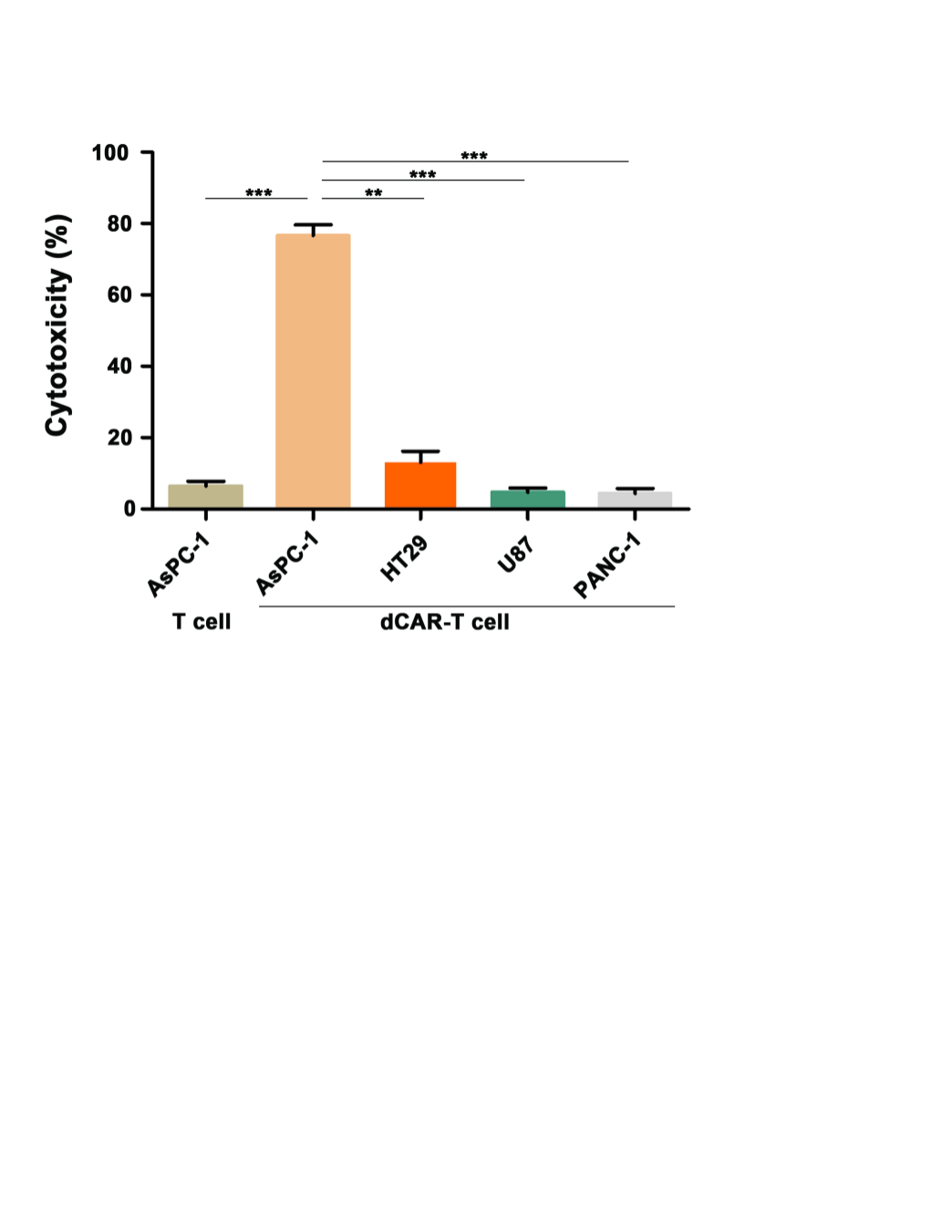


**Figure S3.** dCAR-mediated activation and costimulation of CD8^+^ T cells facilitates significant cytotoxicity and specific activity. dCAR-T cells incubated with AsPC-1 cells expressing CEA and MSLN could achieve remarkable target cell killing. However, data showed that dCAR-T cells did not exert significant cytotoxicity in the presence of single-antigen expressing tumor cells (HT29 cells and U87 cells) or dual-antigen negative tumor cells (PANC-1 cells), which was similar to that of wild T cells. (n = 3, error bars denote standard deviation.)


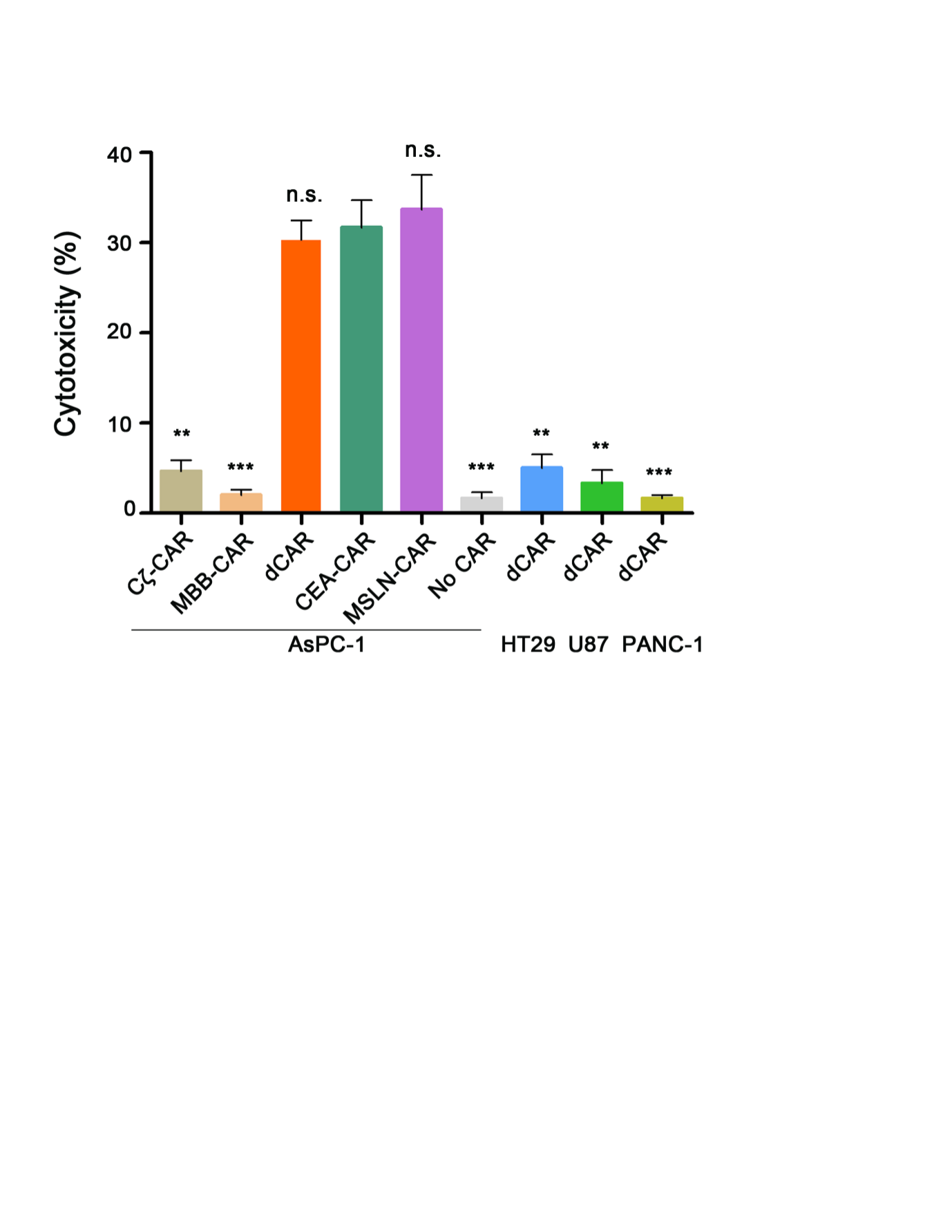


**Figure S4.** dCAR-engineered CD4^+^ T cells could yield slight cytotoxicity compared with CAR-modified CD8^+^ T cells. After an overnight of incubation, dCAR-modified CD4^+^ T cells also eliminate the cognate tumor cells (AsPC-1) (approximately 30% of target cell apoptosis). Therefore, dCAR-CD4^+^ T cells could exert lower cytotoxicity against AsPC-1 cells relative to that of dCAR-CD8^+^ T cells. (n = 3, error bars denote standard deviation.)
